# 6′-*O*-Lactose Ester Surfactants as an Innovative Opportunity in the Pharmaceutical Field: From Synthetic Methods to Biological Applications

**DOI:** 10.3390/ph14121306

**Published:** 2021-12-15

**Authors:** Michele Verboni, Simone Lucarini, Andrea Duranti

**Affiliations:** Department of Biomolecular Sciences, University of Urbino Carlo Bo, 61029 Urbino, Italy; m.verboni@campus.uniurb.it (M.V.); andrea.duranti@uniurb.it (A.D.)

**Keywords:** glycolipids, lactose monoesters, enzymatic synthesis, sugar-based surfactants, fatty acids, permeability enhancers

## Abstract

Glycolipid surfactants are biocompatible and biodegradable compounds characterized by potential applications in various sectors including pharmaceuticals, cosmetics, agriculture, and food production. A specific overview regarding synthetic methodologies and properties of 6′-lactose-based surfactants is presented herein, particularly all the synthetic approaches to this class of lactose esters, such as enzymatic and traditional organic syntheses. Moreover, detailed descriptions of physicochemical data and biocompatibility properties of these molecules, that is, surface tension, critical micelle concentration, emulsifying ability, foaming, particle size distribution, biocompatibility, and safety, are described. Biological applications with a focus on permeability enhancing, antimicrobial activity, and antibiofilm properties of 6′-lactose-based esters are also reported.

## 1. Introduction

**Figure 1:**
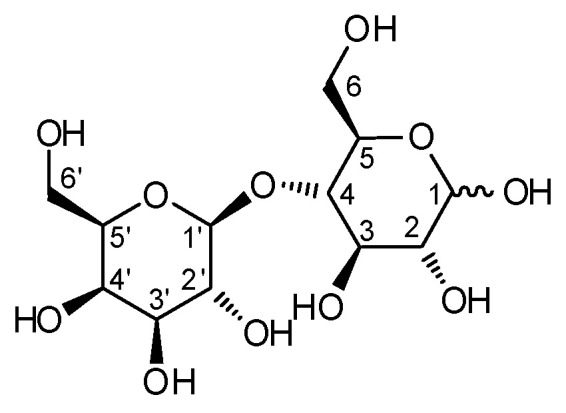
Chemical structure of lactose.

Lactose is a natural disaccharide composed of d-galactose and d-glucose sugars through a β-1,4-glysosidic bond (Figure 1) and constitutes a fundamental nutrient in mammalian milk.

Its importance, however, is also related to the uses of its derivatives, which mainly involve the food (whey, milk powder, confectionery products) and pharmaceutical (excipient in some pharmaceutical forms) industries. In particular, the use of derivatives consisting of fatty acid chains linked to the sugar by an ester function has become increasingly widespread. These molecules, known as sugar-based fatty acid esters or glycolipids, are non-ionic surfactants because of the presence of a hydrophilic polar head (monosaccharides; disaccharides; or, more rarely, oligosaccharides) and a hydrophobic tail (saturated or unsaturated carbon chains). These amphiphilic compounds are of great interest for their multiple uses in the cosmetic, food, and pharmaceutical industries. In fact, in comparison with other surfactants, they are biodegradable, non-toxic, non-irritant, and odourless, and possess a safe biocompatibility profile [1]. Thus, they represent a suitable alternative to the polysorbates in commercial formulations of biologics because they do not induce progressive protein degradation or increased immunogenicity during manufacturing or storage time prior to administration. Specifically, areas dedicated to improving the quality and preservation of food have attracted great interest in recent decades. In fact, glycolipids are considered possible candidates as antimicrobials against biofilm formation by foodborne pathogenic bacteria, preservatives (given their antimicrobial properties), and emulsifying agents, which all increase the stability of foods, such as creams, butter, frozen products, and doughs, thus representing a valid alternative to parabens, often associated with local or systemic adverse reactions. In addition, in agriculture, the use of glycolipids to counteract the contamination of plants by larvae, fungi, and insects has proven to be more advantageous than traditional pesticides owing to the lower toxicity in humans and lesser damage to the environment. In addition, they have recently been explored as permeability enhancers in biologics to improve the absorption of macromolecular drugs across the epithelia.

Glycolipids can be easily obtained in large quantities from inexpensive raw materials such as oily by-products and industrial waste, as well as from microorganisms that optimize the fermentation process. In general, the yields of these bioconversion processes are so high that they allow the retention of huge amounts of glycolipids for various applications [2]. They can also be achieved starting from substrates of natural origin (e.g., lactose from milk and derivatives or sucrose from beet) by means of traditional chemical reactions or catalyzed by enzymes. The enzymatic synthesis presents different selectivity and yields from the substrates depending on the type of enzyme, and offers advantages such as a wide choice of enzymes. This approach allows the formation of the desired ester bond selectively on the primary hydroxyl group of the opportune sugar without further protection and deprotection steps, and the possibility to operate in mild and eco-compatible conditions. With regard to traditional chemical synthesis processes, it is possible to obtain a great variety of glycolipids without the limit of selectivity towards the substrates and thus allow the formation of sugar-diverse chemical libraries. However, it is difficult to selectively obtain the ester bond on the desired hydroxyl of the sugar and the use of protection and deprotection measures not only increases the costs of the process, but also a real decrease in green processes. Furthermore, the use of high temperatures and catalytic acids in the reaction conditions could lead to the formation of toxic, allergenic, and potentially carcinogenic by-products. Consequently, both convenient enzymatic and chemical synthetic strategies requiring less drastic reaction conditions and leading to the formation of regioselective products with high yields have been developed in recent years [3,4].

Among all sugar-based surfactants, sucrose and glucose esters are the most studied and applied derivatives, while lactose esters have received less attention, despite studies showing their potential [5].

In this report, synthetic procedures, physicochemical and biocompatibility properties, and biological applications regarding lactose-6′-*O*-esters are considered.

## 2. Synthetic Procedures

**Figure 2:**
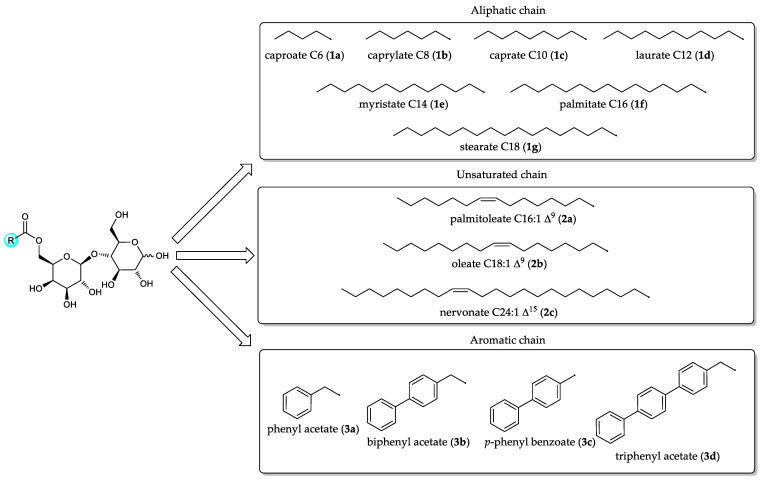
Chemical structure of 6′-lactose-based esters.

The known 6′-*O*-acyl(aryl)lactose esters are obtained from various aliphatic fatty acids (saturated, unsaturated) or aromatic acids and are represented in Figure 2.

Sugar-based esters surfactants offer the advantage of being synthesizable from sustainable and renewable components (fatty acids and carbohydrate) through simple chemical or enzymatic reactions. However, the presence of undesirable products, such as isomers of sugar esters or polyalkylated sugars, is a significant problem resulting from the need to use high temperatures, acid and basic catalysts, and substances with characteristics of activated acyl donors [6]. Therefore 6′-*O*-lactose fatty acids esters are preferentially synthesized by enzyme-catalyzed reactions. Lipases are characterized by stability in non-aqueous environments and green properties and are the enzymes most utilized to carry out regioselective acylation reactions. In fact, the stability in various conditions (e.g., solvents, pH, and temperatures) and the possibility of using an immobilization system involving resins or other approaches, which allow a reuse of the enzyme without significant loss of activity, favor their use [7].

**Scheme 1:**
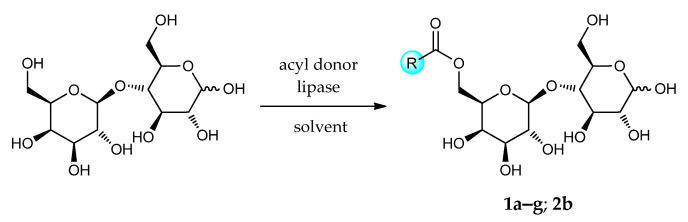
General enzyme-catalyzed regioselective synthetic procedure for 6′-*O*-lactose-based esters.

All of the reported lipases primarily catalyzed esterification of lactose at the C6′-hydroxyl position (Table 1). They allow the esterification reaction directly from the appropriate fatty acid or through the use of vinyl esters, which allow a transesterification reaction to be carried out (Scheme 1). The temperature range generally oscillates between 40 °C and 60 °C, a characteristic interval of the immobilized form, which usually guarantees better conditions because it is more stable than the free form. Some of these 6′-lactose esters were synthesized employing green solvents such as acetone [8,9].

**Scheme 2:**
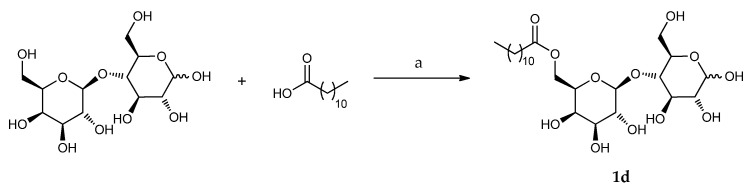
Synthetic procedure for 6′-lactose C12 in organic solvents using activated zeolite as a catalyst. Reagents and conditions: (a) aluminosilicate zeolite, organic solvent, 55 °C, 10 d.

The biggest drawback of the lipase is its high price. Zeolites could represent a valid alternative to expensive lipases. They are aluminosilicate minerals acting as Lewis acid catalysts, available at low cost and preparable in high quantities even from waste materials, such as mollusk shells and eggshells [14,15]. Moreover, they also work as a molecular sieve for water adsorption from the reaction media, promoting the ester production. Enayati et al. have studied aluminosilicate zeolite as a Lewis catalyst for the synthesis of 6′-lactose laurate (C12) using various conditions [16]; the best result (conversion 92%) was obtained for pure lactose at a 1:2 ratio with lauric acid using *tert*-butanol as a solvent and carrying out the reaction for 10 days (Scheme 2).

**Scheme 3:**
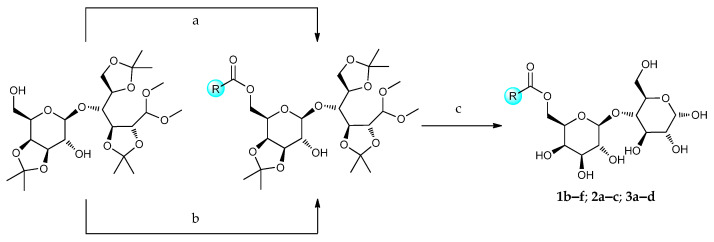
Synthetic procedure for 6′-lactose aliphatic, monounsaturated, and aromatic esters starting from lactose tetraacetate (LTA). Reagents and conditions: (a) aliphatic fatty acid or monounsaturated fatty acid, Lipozyme^®^, toluene, 75 °C, 12 h; (b) aromatic acyl chloride, DIPEA, dry CH_2_Cl_2_, 0 °C, 1 h then rt, 16 h; (c) HBF_4_/Et_2_O, CH_3_CN, 30 °C, 3 h.

Lucarini et al. have synthetized a series of 6′-lactose esters by a two-step procedure (Scheme 3) starting from lactose tetra acetal (LTA) coupled with the opportune aliphatic poly(un)saturated fatty acids or aromatic acids. Lactose aliphatic and poly(un)saturated esters were obtained by enzymatic esterification using Lipozyme^®^ (pathway “a”), followed by acidic deprotection [17,18,19,20]. On the other hand, 6′-lactose aromatic esters were synthesized using acyl chlorides with a classic esterification method (pathway “b”) because lipases do not tolerate aromatic acid substrates [20]. Notably, neither 2′-lactose aromatic esters nor diester derivatives were produced by that chemical esterification.

## 3. Physicochemical and Biocompatibility Properties

Data relating to molecular weight and relevant physicochemical properties, such as hydrophilic–lipophilic balance (HLB) and octanol–water portion coefficient (logP), are reported in Table 2. Owing to the HLB calculated values (range 8.6–13.5), all the compounds are classified as hydrophilic surfactants and act as oil-in-water emulsifiers. However, logP have both negative and positive values with a range of −2.53–3.30, apart from the nervonic acid derivative value, which is affected by the significant elongation of the carbon chain.

With regard to another important property usually taken into consideration, that is, topological polar surface area (TPSA), it should be noted that the characteristics of the compounds analyzed do not affect the value, which was calculated as 195.6 for all compounds (cTPSA, by OSIRIS Property Explorer) [22].

### 3.1. Surface Active Properties (Surface Tension (σ) and Critical Micelle Concentration (CMC))

Surface tension is the tendency of fluid surfaces to shrink to the smallest possible surface area, whereas CMC is defined as the concentration at which a certain number of monomers aggregate to give rise to the formation of micelles. Surface tension at CMC is expressed as γ_CMC_. Both are very important characteristics studied with surfactant glycolipids, in particular those related to lactose derivatives. Lactose-6′-*O*-fatty acid monoesters can be observed in several studies [10,11,13,18,19]. Zhang et al. published that, in the short series of caprylate (C8), caprate (C10), and laurate (C12) esters, chain length was the most important factor influencing the surface properties, whereas the degree of esterification and hydrophilic groups showed little influence [11]. Specifically, values of γ_CMC_ decreased and those of CMC (and HLB) increased when the carbon chain length decreased (C12: γ_CMC_ = 33.06, CMC = 0.31, HLB = 13.1; C10: γ_CMC_ = 31.59, CMC = 0.56, HLB = 13.8; C8: γ_CMC_ = 29.73, CMC = 0.76, HLB = 14.5) [11]. On the other hand, similar studies have been carried out recently by other authors, extending the series of derivatives up to stearate (C18). In particular, Enayati et al. studied surface-active properties such as CMC, σ, interfacial tension, and contact angles for C8, C12, and palmitate (C16) 6′-lactose derivatives, demonstrating a decrease in σ and interfacial tension for all the compounds compared with pure water [10]. Moreover, C6–16 and C18 surfactants displayed a CMC correlated with the length of the hydrophobic side chain (1258–12 μM values) and a low interfacial tension within the oil–water system, whereas there are no clear correlations with the length of the associated side chains in the case of γ_CMC_, measured with myristate (C14)–C18 compounds [13]. In another study, it was possible to observe the relevant influence of the carbon chain length on the surface properties of the amphiphiles and to confirm the inverse relationship between the length chain and CMC of the surfactants (C10–C16) [19]. In the same year of the last three studies, CMC for oleate derivative (C18:1 Δ^9^) was measured by dynamic light scattering (DLS) [18].

### 3.2. Emulsion Properties

Emulsion and emulsion stability are important because of the various potential applications of the examined molecules, such as in food production. With regard to the series of 6′-lactose C8–C12 derivatives in a soybean oil–water system, the emulsifying ability was directly proportional to the increase in the length of the chain (C12 > C10 > C8) and with a good emulsion stability (C12 > C8 > C10) [11]. The importance of intermediate values in the chain was demonstrated by the measurement of the emulsion stability index (ESI) at 0.5% *w*/*w*, through which it was revealed that, in the series C6–C18, a drastic decrease (ESI C16: 797, C18: 309) follows after a progressive increase in the value up to the derivative C14 (ESI C6: 167, C14: 1897) [13].

### 3.3. Foaming Properties

The degree and mode of the dispersion and the nature of the liquid influence the properties of the foam, the development of which is related to the rate of dilating surface at the formation of the bubbles. This phenomenon can be stabilized by surfactants, depending on the time of their adsorption. In the series with 6′-lactose C8–C12 compounds, the foamability varied with the concentration of monoesters and the hydrophobic moiety chain length, with C12 showing the best result in comparison with the others (e.g., at 0.5 g/L C12: 117.3 ± 1.9, C10: 57.3 ± 7.1, C8: 22.0 ± 1.3) [11]. If the series is extended (C6–C18), the propensity for the best result determined by the intermediate chains was confirmed as reported in [13], where a positive correlation between foamability and sample concentration was demonstrated. 6′-Lactose C10 and C12 compounds are the best for both foaming and foaming stability (e.g., C10: 94%, 84%, and 82% over 10′, 20′, and 30′, respectively). The results obtained confirm that foaming properties depend on various factors, such as the absorption capacity of the surfactant at the air/water interface, the rheological properties, and the degree of diffusion of the gas captured in the foam [13].

### 3.4. Particle Size Distributions

This analysis can be used to deeply investigate the evaluation of the emulsifying properties, as carried out by Liang et al. with the lactose derivative C14 in fresh coconut milk [13]. The data obtained show the ability of that mixture to reduce particle size in comparison with the results without C14, with the diameter of particles varying from 0 to 500 μM versus up to 3000 μM, respectively. Evidence of non-uniform dispersion and aggregations is also to be considered in the case of fresh coconut milk dispersion only. However, the reduced particle distribution due to the presence of C14 contributes to making the emulsion more stable.

### 3.5. Thermal Analysis

The stability of the glycolipids is very important, in particular when considering both the role that these substances play as food emulsifiers [13] and studies related to solid dispersions to increase the dissolution rate of hydrophobic drugs [18]. The thermogravimetric analysis (temperature range 50–350 °C) proposed in the paper by Liang et al. with C6–C18 6′-lactose derivatives highlighted a good general stability that was not influenced by the length of the side chain [13]. On the other hand, a differential scanning calorimetry analysis conducted with C18:1 indicated a variation in the heat capacity of this surfactant in the solid state over temperatures, denoting the amorphous state of the molecule studied [18].

### 3.6. Biocompatibility and Safety

The biocompatibility and safety of a surfactant are crucial for the extension of its applications. From a general point of view, 6′-*O*-lactose esters are well tolerated by human cell lines at a relatively high concentration, as demonstrated in several papers. In detail, saturated fatty acid side chain esters C6–C12 showed no appreciable cytotoxicity (IC_50_ > 250 μM) over five human cell lines (MCF-7, A549, H1229, HEPG2-DOX, and AC-16) [13]. On the other hand, surfactants bearing a longer fatty acid side chain (C14–C18) displayed some inhibitory effects on the viability of the human cell lines (69 μM > IC_50_ > 232 μM). In the present series of lactose surfactants, a clear relationship between the variation in the fatty acid side chain and cytotoxicity was shown; in fact, following the elongation of the chain, there is a greater cytotoxicity (expressed by lower IC_50_ values). However, even the IC_50_ values of compounds C14–C18 are significantly higher than their reference counterpart CMCs, thus implying that they could serve as relatively safe surfactants. Similar results were obtained by Lucarini et al. for the same compounds over two different human cell lines (Caco-2 intestinal and Calu-3 airway epithelia), where the cytotoxicity of the compounds, assessed by CellTox^TM^, MTT, and LDH assays, was generally present at greater concentrations than their corresponding CMCs [19]. The authors also investigated the toxicity of the C10–C16 surfactants at high concentrations, demonstrating that the effect is associated with depolarizing mitochondrial membrane potential, increasing nuclear membrane permeability, activation of effector caspases, and apoptosis [19]. On the other hand, monounsaturated fatty acid side chain lactose esters palmitoleate (C16:1), oleate (C18:1), and nervonate (C24:1) showed relatively low cytotoxicity. In particular, C18:1 presents an IC_50_ value of about 230 μM for Caco-2 cell line calculated by MTS assay [18]. However, C16:1 and C24:1 did not display notable toxicity (cell viability > 80%) to Caco-2 cells, regardless of the applied concentrations (up to 1000 μM) [17]. It then seems that monounsaturated fatty acid side chain esters C16:1 and C18:1 are less cytotoxic compared with their 6′-*O*-lactose saturated derivatives (C16 and C18, respectively). With regard to the biocompatibility and safety of aromatic-lactose esters, only one surfactant is reported in the literature, that is, lactose biphenylacetate (Bpa) did not show appreciable toxicity to Caco-2 cells by MTS assay (cell viability > 90% with a 500 μM concentration) [20].

Sugar esters are usually considered perfectly safe for use in the food, cosmetics, and pharmaceutical industries, because they are in vivo converted into harmless carbohydrates and fatty acids. However, the cytotoxicities of sugar esters arise from the compounds themselves and not their metabolic derivatives and constituents. Similarly, the 6′-*O*-lactose esters reported herein follow the biocompatibility and safety of the sugar esters’ class and could be considered safe surfactants for widespread applications. A possible limitation in their use could be lactose intolerance in humans, a factor that varies significantly among different racial groups.

## 4. Biological Applications

The intrinsic characteristics of sugar-based surfactant compounds, that is, being amphiphilic, biodegradable, non-toxic, non-irritant, tasteless, and odorless molecules, are very promising for an extensive use in food, pharmaceutical, and cosmetic industries. Herein, the most important applications in the biological field will be discussed.

### 4.1. Permeability Enhancing

Sugar-based esters can be used as permeability enhancers in drug delivery, offering a suitable non-invasive alternative route of administration, such as oral or pulmonary, to others (e.g., injection). The permeability enhancing activity of 6′-lactose aliphatic saturated [19] and unsaturated [17,18] fatty acid esters has been reported. Trans-epithelial electrical resistance (TEER) experiments were conducted to demonstrate the increasing effect on membrane perturbation associated with their toxicological profile. With regard to this, a desired reversible effect on the TEER is correlated to transient modulation of tight junction opening, while a non-reversible effect is due to a permanent perturbation of membrane integrity. Lucarini et al. performed TEER studies on Calu-3 and Caco-2 monolayer cells of 6′-lactose aliphatic (C10-C16) derivates at the IC_50_ calculated from 3-(4,5-dimethylthiazol-2-yl)-2,5-diphenyltetrazolium bromide (MTT) assay and at the highest concentration, which shows 100% viability in MTT assay [19]. Therefore, TEER measured for each lactose ester was correlated with the corresponding concentration and with the lipophilic chain length. 6′-Lactose laurate (C12) has a good balance between safety and efficacy thanks to its ability to lower TEER on Calu-3 significantly both at the lowest (0.476 mM; 0.025% *w/v*) and at the highest (1.069 mM; 0.056% *w/v*) concentrations. On the contrary, the other lactose esters exhibit a slight reduction in TEER. Recently, McCartney et al. reported the permeability enhancing effect of C12 across isolated rat intestinal mucosae [23]. In fact, C12 causes a significant fluidization of the Caco-2 plasma membrane at 0.5 mM (above CMC) within 1 min of exposure with a decreasing generalized polarization to 0.174 ± 0.006 (Laudren assay), and an increase in the [^14^C] mannitol P_app_ (apparent permeability coefficient) was observed at concentrations > 1 mM (*p* < 0.01). This rise is inversely correlated with the decreasing TEER effect. C12-lactose showed a better permeability enhancing effect than C12-trehalose ester and comparable results with the commercially available C12-sucrose ester [23]. TEER was also measured for 6′-lactose monounsaturated esters C16:1, C24:1 [17], and C18:1 [18] in Caco-2 cell monolayer. Concentrations of these unsaturated surfactants selected from the cytotoxicity assays up to the IC_50_ values were applied and a dose–effect relation for TEER was observed. For C16:1, TEER values sharply decrease, with maximal lowering by 62–68% of the baseline value after 2.5 h. However, C24:1 leads to a gradual decrease in TEER, and a minimal value was observed after 3 h (41–65%). For C18:1, the maximum decrease in TEER was observed after 120 min, with TEER remaining stable from that point and up to 180 min subsequent application of the compound. TEER values at 120 min were roughly 70% (0.06 mg/mL), 55% (0.125 mg/mL), and 40% (0.25 mg/mL) of the baseline value. TEER recovered after 24 h, emphasizing the reversible effect of the lactose-unsaturated fatty acid monoesters on tight junction openings. The effect of C16:1 and C24:1 on the permeability of Fitc-ovalbumin (45 Kda) as model protein across Caco-2 cell monolayer was also studied [17]. At 0.2 mg/mL, C16:1 enhanced the FITC-labelled ovalbumin permeability by 11.5-fold when compared with the control. On the other hand, C24:1 at 0.1 mg/mL increased 2.5-fold FITC-OVA permeability. C18:1 was also tested for its ability to improve the in vitro permeability of Fitc-labelled dextran (4 Kda) as a model macromolecular drug. The observed apparent permeability was found to be dependent on the applied surfactant concentration, which reached 7.7-fold enhancement at the maximum concentration (0.41 mM, 0.25 mg/mL). Overall, the results suggest the involvement of the intercellular tight junction opening (paracellular route) in improving permeability of biologics in the presence of 6′-lactose esters at a non-toxic concentration. Nevertheless, other mechanisms cannot be excluded.

### 4.2. Antimicrobial Activity

Various sugar derivatives have been proposed as safe and efficient preservatives, particularly to replace parabens in the pharmaceutical, cosmetic, and food fields. In this context, the antibacterial activities of various lactose fatty acid esters against several different food-borne and human pathogens, gram positive, gram negative, and fungi, were measured by minimum inhibitory concentration (MIC) values [11,12,13,17,18,20,24,25] (Table 3). Generally, 6′-lactose medium-length chain esters show more microbial inhibition than those with shorter or longer saturated chains [12,13,20]. 6′-Lactose C10 and C12 were the most active saturated surfactants against a broad spectrum of food-borne pathogens [20]; therefore, C14 [12] is less effective than these. However, C14 was found to be the best saturated compound of the series versus *E. faecalis* ATCC 29212, showing the same MIC value as sodium benzoate (64 μg/mL) [13]. The antibacterial activity of reported 6′-lactose esters decreased with the increasing length of the fatty acid saturated chain. In fact, C18, as well as derivatives with a very short hydrocarbon tail like C6, did not show any inhibition against tested microorganisms [13].

Nevertheless, the best antimicrobial effect was observed with 6′-lactose unsaturated fatty acid esters [17,18]. In particular, C16:1, C18:1, and C24:1 proved to be more effective against numerous pathogens, with MIC values between 64 and 128 μg/mL for yeast and both gram-positive and gram-negative bacteria compared with the parabens (>1024 μg/mL) [17,18]. C16:1, C18:1, and C24:1 showed comparable activity against the panel of bacteria compared with gentamicin, although slightly less active [17,18]. In particular, C16:1 and C24:1 were more active against *E. coli* O157:H7 ATCC 35150 strain with respect to the same reference drug (MICs 64 μg/mL vs. 128 μg/mL, respectively) [17]. The antimicrobial activities of 6′-lactose based esters reported in Table 3 are generally comparable to other sugar based surfactants, such as alkyl maltosides [26]. The few known aryl or alkyl aromatic lactose-based surfactants showed comparable MIC values to the corresponding saturated medium-length chain (C8–C12) (256 μg/mL) [20]. Aryl or alkyl aromatic lactose esters, together with the other lactose-based surfactants analyzed therein, could be an interesting, safe, and biocompatible antibacterial additive alternative to sodium benzoate or toxic parabens in the cosmetics industry. Indeed, most of the reported amphiphilic compounds showed an antibacterial effect at non-toxic concentrations for Calu-3 and Caco-2 cell lines. MICs were found at 256 μg/mL for all the tested microorganisms by Campana et al. [20], except for *S. aureus* ATCC 43387 and *P. aeruginosa* ATCC 9027, for which a higher concentration was measured.

### 4.3. Antibiofilm Properties

A very recent promising tool in the armamentarium of biological applications is the possibility that some surfactants counteract the formation of bacteria biofilms. In fact, two 6′-lactose derivatives, aliphatic C10 and aromatic Bpa, were able to do this during the experiments carried out after a 24 h, 48 h, or 96 h incubation and with selected bacteria (*Escherichia coli*, *Listeria monocytogenes*, *Staphylococcus aureus*, and *Staphylococcus enteritidis*) [20]. In particular, C10 showed a good activity after 96 h (*S. aureus*, 2× MIC 55%), whereas Bpa was the best, being active after 24 h, 48 h, and 96 h with MIC and 2× MIC. In the last case, the best result was obtained with *E. coli* after 96 h (MIC 85%, 2× MIC 92%), but good/consistent antibiofilm properties were shown after 24 h (MIC 40%, 2× MIC 47%) and 48 h (MIC 61%, 2× MIC 66%) or versus other bacteria (e.g., 96 h: *L. monocytogenes*, MIC 76%, 2× MIC 79%; *S. aureus*, MIC 33%, 2× MIC 54%; *S. enteritidis*, MIC 48%, 2× MIC 53%) [20]. Overall, the data suggest that the presence of the tested molecules in the culture medium limits the initial formation of the biofilm and continues to interfere with its subsequent maturation [20].

## 5. Conclusions

Lactose-based surfactants are an emerging broad group of biocompatible and biodegradable compounds with established and potential future applications in the pharmaceutical, biomedical, cosmetic, and food industries. Specifically, this report focuses on 6′-*O*-lactose monoester derivatives, highlighting their main synthetic procedures, physicochemical and biocompatibility properties, and the most interesting biological applications.

## Figures and Tables

**Table 1:** Enzymatic synthesis of 6′-*O*-acyllactose esters starting from lactose.

Immobilized Lipase	Type	Acyl Donor	Solvent	Molar Ratio Lactose/Acyl Donor	Drying Agent	T (°C)	Time (h)	Yield (%)	Refs.	#
**Novozyme 435**	*Candida antharctica*	Vinyl laurate	2M2B	1:3	MS 3 Å	55	24–72	22	[8]	**1d**
**Novozyme 435**	*Candida antharctica*	Caprylic acid	*n*-Hexanes	1:1	MS 4 Å	55	12	80	[10]	**1b**
		Lauric acid				50	10	93		**1d**
		Palmitic acid				55	12	97		**1f**
**NER-CRL**	*Candida rugosa*	Capric acid	Acetone	2:1	MS	50	48	70	[9]	**1c**
**Novozyme**	*Thermomyces lanuginous*	Vinyl laurate	Acetone	1:3	MS 3 Å	55	24–72	43	[8]	**1d**
**Lipozyme^®^ TL IM**	*Thermomyces lanuginous*	Vinyl caprylate	2M2B/ DMSO (4:1)	1:10	MS 4 Å	50	4	nr	[11]	**1b**
		Vinyl caprate								**1c**
		Vinyl laurate								**1d**
**TM2**	*Thermomyces lanuginous*	Vinyl caprylate	2M2B	1:2.1	MS	60	48	nr	[12]	**1b**
		Vinyl caprate								**1c**
		Vinyl laurate								**1d**
		Vinyl myristate								**1e**
**Lipozyme^®^ TL IM**	*Thermomyces lanuginous*	Vinyl caproate	dry THF/Py (1:1)	1:3	–	55	48	76	[13]	**1a**
		Vinyl caprylate						71		**1b**
		Vinyl caprate						65		**1c**
		Vinyl laurate						63		**1d**
		Vinyl myristate						78		**1e**
		Vinyl palmitate						71		**1f**
		Vinyl stearate						58		**1g**
**Amano Lipase PS-C I**	*Pseudomonas cepacia*	Vinyl laurate	2M2B	1:3	MS 3 Å	55	24–72	57	[8]	**1d**
**Lipozyme^®^**	*Mucor miehei*	Vinyl laurate	2M2B	1:3	MS 3 Å	55	24–72	52	[8]	**1d**

2M2B = 2-methyl-2-butanol; DMSO = dimethyl sulfoxide; THF = tetrahydrofuran; Py = pyridine; MS = molecular sieves; nr = not reported.

**Table 2:** Calculated physicochemical properties of the sugar-based ester surfactants.

Compound	Lactose Ester	MW	HLB ^a^	LogP ^b^
**1a**	Caproate C6	440.4	13.5	−2.16
**1b**	Caprylate C8	468.5	12.7	−1.25
**1c**	Caprate C10	496.6	12.0	−0.34
**1d**	Laurate C12	524.6	11.4	0.57
**1e**	Myristate C14	552.7	10.8	1.48
**1f**	Palmitate C16	580.7	10.3	2.39
**1g**	Stearate C18	608.8	9.8	3.30
**2a**	Palmitoleate C16:1 Δ^9^	578.7	10.3	2.14
**2b**	Oleate C18:1 Δ^9^	606.7	9.8	3.04
**2c**	Nervonate C24:1 Δ^15^	690.9	8.6	5.77
**3a**	Phenylacetate (Pa)	460.4	12.9	−2.53
**3b**	Biphenylacetate (Bpa)	536.5	11.1	−0.87
**3c**	*p*-Phenylbenzoate (*p*-Pb)	522.5	11.4	−0.87
**3d**	Triphenylacetate (Tpa)	612.6	9.7	0.79

^a^ Calculated HLB by Griffin’s method for non-ionic surfactants [21] (HLB = 20 × (MW hydrophilic portion/MW)). ^b^ Calculated octanol-water portion coefficient LogP (by OSIRIS Property Explorer) [22].

**Table 3:** Minimum inhibitory concentration values of 6′-*O*-lactose esters ^a^.

Microorganism	C6 1a	C8 1b	C10 1c	C12 1d	C14 1e	C16 1f	C18 1g	C16(Δ^9^) 2a	C18(Δ^9^) 2b	C24(Δ^15^) 2c	Pa 3a	Bpa 3b	p-Pb 3c	Tpa 3d
***B. cereus* 13061**		<5000	<3000	<1000	<1000									
***E. faecalis* ATCC 29212**	Na ^b^	256	128	256	64	>256	na	64	128	64	256	256	256	256
***E. faecalis* V538**		na	<5000	<1000	na									
***L. monocytogenes* ATCC7644**		256	256	>256	>256	>256		64	256	64	256	256	256	256
***L. monocytogenes* EGDe**		<3000	<3000	<100	na									
***M. KMS* NA**		na	<1000	<50	<5000									
***S. aureus* ATCC 25923**	na	na	>512	>512	na	na	na							
***S. aureus* (MRSA) ATCC 43300**	na	na	na	>512	>512	na	na							
***S. aureus* ATCC 43387**		256	256	>256	>256	>256		128	128	128	>256	256	256	256
***S. aureus* CICC 21600**		>4000	4000	500										
***S. mutans* FSL R2-**		na	na	<1000	na									
***S. suis* 89/1591**		<3000	<3000	<1000	<3000									
***E. coli* ATCC 25922**	na	na	na	>512	na	na	na							
***E. coli O157:H7* ATCC 35150**		256	256	>256	>256	>256		64	128	64	256	256	256	256
***K. pneumoniae* ATCC 13883**		256	256	>256	>256	>256					256	256	256	256
***P. aeruginosa* ATCC 27853**	na	na	>512	>512	>512	na	na							
***P. aeruginosa* ATCC 9027**		256	256	>256	>256	>256		128	128	128	>256	256	256	256
***S. enterica* CMCC 50094**	na	>512	na	>512	>512	na	na							
***S. enteritidis* ATCC 13076**		256	256	>256	>256	>256		128	128	64	256	256	256	256
***Y. enterocolitica* ATCC 27729**								64	128	64				
***C. albicans* ATCC 10231**		256	128	256	256	256		64	128	64	256	256	256	256

^a^ The values are expressed as μg/mL. ^b^ na = not active.

## Data Availability

Data sharing not applicable.
